# Yes, The Government Should Tax Soft Drinks: Findings from a Citizens’ Jury in Australia

**DOI:** 10.3390/ijerph110302456

**Published:** 2014-02-27

**Authors:** Nicole Moretto, Elizabeth Kendall, Jennifer Whitty, Joshua Byrnes, Andrew P. Hills, Louisa Gordon, Erika Turkstra, Paul Scuffham, Tracy Comans

**Affiliations:** 1 Population and Social Health Research Program, Griffith Health Institute, Griffith University, Meadowbrook QLD 4131, Australia; E-Mails: n.moretto@griffith.edu.au (N.M.); e.kendall@griffith.edu.au (E.K.); j.byrnes@griffith.edu.au (J.B.); louisa.gordon@griffith.edu.au (L.G.); e.turkstra@griffith.edu.au (E.T.); p.scuffham@griffith.edu.au (P.S.); 2 Centre for Applied Health Economics, School of Medicine, Griffith University, Meadowbrook QLD 4131, Australia; 3 School of Pharmacy, The University of Queensland, Woolloongabba QLD 4102, Australia; E-Mail: j.whitty@uq.edu.au; 4 Centre for Nutrition and Exercise, Mater Research Institute, University of Queensland, Aubigny Place, South Brisbane QLD 4101, Australia; E-Mail: ahills@mmri.mater.org.au; 5 Centre for Musculoskeletal Research, Griffith Health Institute, Griffith University, Gold Coast QLD 4222, Australia; E-Mail: a.hills@griffith.edu.au

**Keywords:** taxation, childhood obesity, sweetened drinks, Citizens’ Jury, public engagement

## Abstract

Taxation has been suggested as a possible preventive strategy to address the serious public health concern of childhood obesity. Understanding the public’s viewpoint on the potential role of taxation is vital to inform policy decisions if they are to be acceptable to the wider community. A Citizens’ Jury is a deliberative method for engaging the public in decision making and can assist in setting policy agendas. A Citizens’ Jury was conducted in Brisbane, Australia in May 2013 to answer the question: Is taxation on food and drinks an acceptable strategy to the public in order to reduce rates of childhood obesity? Citizens were randomly selected from the electoral roll and invited to participate. Thirteen members were purposively sampled from those expressing interest to broadly reflect the diversity of the Australian public. Over two days, participants were presented with evidence on the topic by experts, were able to question witnesses and deliberate on the evidence. The jurors unanimously supported taxation on sugar-sweetened drinks but generally did not support taxation on processed meats, snack foods and foods eaten/ purchased outside the home. They also supported taxation on snack foods on the condition that traffic light labelling was also introduced. Though they were not specifically asked to deliberate strategies outside of taxation, the jurors strongly recommended more nutritional information on all food packaging using the traffic light and teaspoon labelling systems for sugar, salt and fat content. The Citizens’ Jury suggests that the general public may support taxation on sugar-sweetened drinks to reduce rates of obesity in children. Regulatory reforms of taxation on sugar-sweetened drinks and improved labelling of nutritional information on product packaging were strongly supported by all members of the jury. These reforms should be considered by governments to prevent childhood obesity and the future burden on society from the consequences of obesity.

## 1. Introduction

Childhood obesity is of particular concern as overweight and obese children have a high chance of becoming obese adults and have an increased risk of later cardio-metabolic morbidity and premature mortality [1]. An increase in the total number of years spent in an obese state increases the risk for cardiovascular, cancer and all-cause mortality, emphasising the increased benefits of targeting the prevention of obesity in children and young people [2]. Importantly, targeting the prevention of obesity in children may be far less expensive than having to treat the consequences of obesity later in life [3].

Current initiatives to improve food choices in Australia have focussed on voluntary measures in the form of self-regulation of television advertising to children and food labelling [4,5,6]. These measures have been shown to be inadequate with deregulated food markets linked to increased fast food transactions and increasing body mass index (BMI) with the sharpest increases occurring in Canada and Australia (1999–2008) [7]. In contrast, countries with stringent food market regulation such as Italy, the Netherlands and Greece had relatively small increases in both fast food consumption and BMI [7]. One regulatory strategy with considerable potential to reduce the consumption of obesogenic foods, and therefore obesity, is taxation [8,9]. Using taxation to increase the price of energy-dense nutrient-poor foods is likely to have an impact of food consumption patterns in Australia. However, it is important to first clearly define what categories of food contribute most to weight gain and identify whether a tax on these items is likely to be practical and acceptable.

In 2010, the Australian government commenced the development of a National Food Plan (NFP) aimed at integrating food-related policies in Australia [10]. The NFP has been criticised as having a strong focus on economic growth and food production rather than on promoting the availability of affordable and nutritious food to help consumers make healthier food choices [11]. The Independent Panel for the Review of Food Labelling Law and Policy presented a report in which it recommended the development a national nutrition policy to establish monitoring and food labelling systems in Australia [12]. Although there is strong support for a National Nutrition Policy [11], some of the key concerns about the policy include the lack of evidence on the effectiveness of the star rating labelling system, the length of time taken to implement the labelling changes and the reliance on food industry to voluntarily implement label changes [13].

Public engagement in policy decisions is increasingly viewed as an essential part of decision making in the health area given that the public are the key stakeholders of any decisions that are made [14]. Members of the public can provide their view of the values and priorities of their community. This allows for both improving the trust and confidence in the health system and ensuring that decisions fit with the ideals of a participatory democracy [15]. Knowing the public viewpoint is also important for policy implementation as Governments are often unwilling to make unpopular decisions, particularly those involving taxes. A Citizens’ Jury is one method of public deliberation that offers a relatively high level of participation by public participants in policy decisions [16,17,18]. It is a well-accepted approach for engaging the public in decision-making on a specific topic, including in the area of health policy [19,20,21,22,23]. As a deliberative form of pubic engagement, it is well suited to investigating public opinion around topics that may be sensitive or divisive, such as the role of taxation in obesity prevention. This paper describes the findings and recommendations of a Citizens’ Jury exploring pubic perspectives on taxation of food and drinks as a preventive strategy for childhood obesity.

## 2. Methods

### 2.1.Study Design

The Citizens’ Jury was convened to assess the likely acceptability of taxation to prevent childhood obesity by influencing the purchasing of obesogenic food and drinks by parents. All aspects of the Citizens’ Jury process including the development of questions, the selection of jurors and expert witnesses, the schedule and content of the Citizens’ Jury and the procedure were based on the standardised Citizens’ Jury methods [19]. The jurors were asked to reach a verdict and make recommendations about taxation as an obesity-prevention strategy based on evidence provided by clinical, policy and academic expert witnesses from a wide range of perspectives. The jurors were able to “cross-examine” the experts who provided evidence and recall “witnesses” to assist them in making their recommendations.

#### 2.1.1. Development of Questions

The questions to be put to the Citizens’ Jury were developed based on information from a literature review and expert panel. The literature review was conducted to examine current patterns of consumption in Australian children and to assess taxation measures on food and drinks in an international context. To supplement the literature and provide guidance in interpretation, a panel of Australian experts on nutrition and obesity was convened. This panel aimed to identify foods and drinks that are associated with overweight and obesity during early childhood and the categories of foods that may be amenable to taxation as a strategy to reduce consumption. The expert panel identified a number of key types of food and drinks that may be contributing to early childhood obesity, including sugar-sweetened drinks, processed meats, portioned snack foods and takeaway foods. The panel reached consensus that prepared foods eaten outside the home, sugar-sweetened drinks and high protein/low nutrient quality infant formula may have the potential to respond to taxation. 

Based on these recommendations, the following five questions were developed and put to the jurors for deliberation at the Citizens’ Jury:

(a) Is taxation an appropriate strategy for reducing childhood obesity amongst 0–5 year olds?;
(b) Is it appropriate to tax sugar-sweetened drinks as a strategy for reducing childhood obesity? (sugar-sweetened drinks refers to all drinks with added sugar including soft drinks (carbonated drinks), cordials, flavoured milks, fruit juices, fruit drinks and vitamin waters);
(c) Is it appropriate to tax processed meats as a strategy for reducing childhood obesity? (processed meats refers to meat and meat alternatives that have been processed including chicken nuggets, sausages and meats with high fat and sodium content);
(d) Is it appropriate to tax snack foods as a strategy for reducing childhood obesity? (snack foods refers to sweet or savoury snack packs and individually wrapped snacks including packets of biscuits, potato chips, sweets, muesli bars, small cakes, muffins and crackers with cheese), and
(e) Is it appropriate to tax food eaten away from home as a strategy for reducing childhood obesity? (foods eaten outside the home refers to takeaway foods that are purchased and/or eaten outside the home including well-known fast food brands and specific items with high energy fat, sugar and sodium content).

Jurors were provided with information on the types of taxes in Australia including personal income tax, business tax, capital gain tax, environmental tax, a value added tax (VAT) also known as Goods and Services Tax (G.S.T.), *ad valorem* tax, excise tax, Pigovian tax and volumetric tax. Jurors were not asked to specify the type of tax that may be suitable to address childhood obesity, but rather were asked to deliberate on whether taxation of food and drinks is appropriate as a general preventive strategy. Infant formula was identified by the expert panel as a food amenable to taxation, however this category of food was not included in the questions posed to the jurors as insufficient evidence was able to be provided for the jurors to make an informed decision on this category of food.

#### 2.1.2. Selection of Jurors

Recruitment of participants in this study was undertaken alongside another unrelated Citizens’ Jury project being conducted at Griffith University. A random sample of 2,000 people living in metropolitan and regional areas of a state health service district in the south-eastern area of Queensland was selected from the Queensland electoral roll and invited to participate in a previous Citizens’ Jury project at Griffith University [24]. A total of 314 respondents who had expressed interest in participating and had not been selected to take part in the previous project were sent a letter of invitation, an information sheet regarding two further research studies (the current study and one other independent study that nevertheless also related to obesity) and a screening questionnaire. The Citizens’ Jury was held on a weekend to increase the response rate and participation from those in the labour force. In addition, jurors were offered a stipend of AUD$200 for sitting on the jury and vouchers to assist with their transportation and accommodation needs to help minimise volunteer bias.

Of the 140 questionnaires (45%) that were returned, a total of 59 respondents expressed their interest and availability in participating in both of the studies (40 respondents), the current study only (nine respondents) and the other study only (10 respondents).

The current study aimed to assemble a minimum of 12 persons to sit on the Citizens’ Jury. Fifteen individuals were purposively selected from this pool to incorporate a range of ages, genders and backgrounds, were contacted by telephone and invited to participate as jurors in the current Citizens’ Jury. Two participants declined the invitation to participate due to a lack of interest in the study and work commitments.

The nominated Citizens’ Jury consisted of 13 members of the public who reflected the diversity of the community. The jurors were eight females (62%) and five males (38%) who ranged in age from 18 years to over 65 years. The 13 jurors were each sent an information sheet and consent form which were completed and returned prior to the commencement of the Citizens’ Jury. The consent form also included the consent for the entire Citizens’ Jury process to be audio recorded. Participants were able to withdraw from the study and leave the Citizens’ Jury at any time.

#### 2.1.3. Selection of Expert Witnesses

Expert witnesses were carefully selected by the project team and recruited from state health departments, hospitals and a state community service organisation within their professional networks in Australia. The expertise of the witnesses is indicated in Table 1. The experts provided jurors with information on key topics from different perspectives.

### 2.2. Procedure

The Citizens’ Jury was conducted in May, 2013 in Brisbane, Queensland, Australia. The jurors were presented with evidence from the expert witnesses on a wide range of topics including childhood obesity epidemiology and treatment, taxation, impact of changing food prices and information on the childhood consumption of sugar-sweetened drinks, snack foods, processed meats and foods eaten/purchased outside the home. The schedule of the Citizens’ Jury and content of the presentations from the expert witnesses are summarised in Table 1.

Two independent facilitators guided the jurors through the Citizens’ Jury process, facilitated deliberative discussions, assisted jurors with complex issues and provided practical support. The jurors were provided with pre-reading material including background information on the research project, childhood obesity and taxation of food and drinks. Jurors were provided with a handbook which contained key information about the Citizens’ Jury process, biographies of the expert witnesses and a list of the key questions for deliberation.

Some members of the project team were present during the presentations from the expert witnesses on Day 1 and were available throughout the Citizens’ Jury to provide practical support to the facilitators and to assist the jurors in clarifying any information as needed. A representative of the funding body was present for the delivery of the jurors’ findings and acceptance of the jury report on Day 2.

**Table 1:** Schedule and content of the Citizens’ Jury.

*Day 1*
Welcome—Independent facilitators The individual introductions of facilitators, project team and jurors followed by an ice-breaker activity.
Introduction—*Research project leader (T.C.)* The topics covered included an overview of: the nature of overconsumption in children; expenditure on healthcare; health prevention and costs associated with obesity; findings from The Australian Diabetes Obesity and Lifestyle Study; food industry and voluntary measures to address obesity; examples of current food labels; educational programs; campaigns and food advertising; examples of taxation on food and drinks in other countries; and information on the regulation of the tobacco industry.
Background of childhood obesity and associated health issues—*Epidemiologist manager, Preventive health unit* The topics covered included: the prevalence of obesity in children and adults in Queensland; rates of childhood obesity in Australia; Body Mass Index and risks associated with excess weight; nutrition and activity levels of children; causes of overweight and obesity; and costs associated with obesity in Queensland.
Overview of taxation—*Health research economist* The topics covered included: background to taxation and types of taxes; taxation and economic welfare; overview of a Pigovian tax; advantages and disadvantages of taxation; and examples of taxation in the alcohol industry.
Session 1 Panel—*Paediatric dietitian and a policy manager, Queensland Council of Social Service* Members of the panel responded to questions from the jurors on the topics of the experience of treating obesity in children and the impact of changing food prices on families.
Session 2 Topic: Snack foods—*Paediatric dietitian* The topics covered included: overview, rates and examples of snack food consumption in children; overview of recommendations from dietary guidelines for children; types of foods that should be limited for children; example of a recommended diet and sample meal plan for young children; issues associated with snack food consumption and obesity in young children; and examples of nutritional content and prices of popular snack foods. Topic: Processed meats—*Senior community dietitian* The topics covered included: overview of processed meats; nutritional content of meat products; information on nitrates and nitrites; Australian Dietary Guidelines regarding foods containing saturated fat and salt; benefits of children consuming unprocessed meats; and rates of consumption of processed meat products in children. Topic: Foods eaten away from home—*Public health nutritionist* The topics covered included: household expenditure of food and drinks; extra foods consumed by children; proportion of evening meals cooked at home *versus* prepared outside of the home; obesity and health issues associated with takeaway foods; example of the nutritional content of takeaway meals for children; example of the prices of takeaway foods with taxation; and issues to consider regarding foods eaten away from the home.
Session 3 Topic: Sugar-sweetened drinks—*Clinical paediatric dietitian/researcher* The topics covered included: overview of the nature of sugar; nutritional content of popular sugar-sweetened drinks; Australian Dietary Guidelines for discretionary serves for children; frequency of soft drink consumption in children; overview of strategies to prevent childhood obesity; and clinical experience treating obesity in children.
Supplementary media sources A position statement from the *Australian Food and Grocery Council* in the form of a radio interview [25] and newspaper article [26] were provided to the jurors along with a position statement from the *Australian Medical Association* in the form of a newspaper article [26]. Discussion and summary of important topics
*Day 2*
Recap of previous day Summary of the discussions from the previous day and discussion of agenda for Day 2.
Deliberations Jurors engaged in two deliberation sessions led by the facilitators. The time for discussions and deliberations remained flexible in order to ensure that the verdicts could be reached.
Verdicts and recommendations The jurors issued the verdicts and recommendations. Two representatives of the jury were appointed to present their findings at a forum which included the project team and a representative of the funding body. The representative of the funding body accepted the report and provided a response. The jurors provided feedback on the Citizens’ Jury process and completed evaluation forms. A final report of the project was completed by the project team and was made available to the jurors and the public.

No members of the project team or funding body were present during the deliberations. The jurors had the opportunity to ask the expert witnesses questions throughout the Citizens’ Jury. The facilitators used flip charts to keep track of the discussions and ideas.

### 2.3. Outcome Measures

#### 2.3.1. Voting Preferences of Jurors

At the commencement of the Citizens’ Jury, each of the five questions were written on separate pieces of paper and hung on the wall. A Visual Analogue Scale (VAS) was displayed alongside each of the questions. The scale was anchored with labels at each end (strongly disagree and strongly agree) and in the centre (neutral or unsure). Each juror was provided with a set of uniquely coloured stickers and was instructed to place a sticker on the scale to represent their level of agreement with the question. The voting was undertaken individually by each of the jurors at the same time and jurors were able to the see the responses of the other jurors. The facilitators and project team were present during the voting. The jurors were asked to vote on each of the questions at three separate time points over the course of the Citizens’ Jury: before the presentations from the expert witnesses (start), at the end of the first day (middle), and at the end of the second day (end).

The voting scales were approximately 70 cm in length. Each of the individual votes from the jurors, as indicated by each uniquely coloured sticker, was measured with a ruler and recorded in centimetres. This data was then converted into scaled scores ranging from 0 (strongly disagree) to 10 (strongly agree). The average was then calculated for all of the questions at each of the three time points; start, middle and end.

#### 2.3.2. Verdicts and Recommendations

The jurors reached verdicts for each of the five questions based on the evidence presented to them and through the deliberative discussions. The jurors developed recommendations relevant to each of the five questions and to the overarching issue of the prevention of childhood obesity in Australia. One of the facilitators transcribed the verdicts and recommendations on a computer with a data projector using the words that were suggested and approved by the jurors. An audio recorder was used to record the Citizens’ Jury process. This paper focuses on the report that was compiled by the jurors which outlines the verdicts and recommendations of the Citizens’ Jury.

## 3. Results and Discussion

### 3.1. Profile of the Jury

Demographic characteristics of the 13 citizens selected to participate in the Citizens’ Jury are presented in Table 2. The jurors broadly reflected the diversity in the community and were from a diverse range of ages, family situations, educational, employment and household income levels.

**Table 2:** Demographic characteristics of the jurors.

Demographic Characteristic of Jurors	N (%)
Gender	
Male	5 (38)
Female	8 (62)
Age	
18–34 years	1 (8)
35–44 years	2 (15)
45–54 years	3 (23)
55–64 years	4 (31)
<65 years	3 (23)
Children under 18 years living at home	
0 children	9 (69)
1 child	1 (8)
2 or more children	3 (23)
Born overseas	5 (38)
Speaks a language other than English at home	0 (0)
Indigenous	0 (0)
Education	
Did not complete high school	2 (15)
Up to year 12	3 (23)
Diploma or trade certificate	7 (54)
Bachelor’s degree or higher	1 (8)
Employment	
Full-time	5 (38)
Part-time	4 (31)
Unemployed	0 (0)
Not in labour force/Retired	4 (31)
Annual household income	
<$42,000	4 (31)
$42,000–$130,000	7 (54)
>$130,000	1 (8)
Not stated	1 (8)

### 3.2. Jury Verdicts

**Figure 1:**
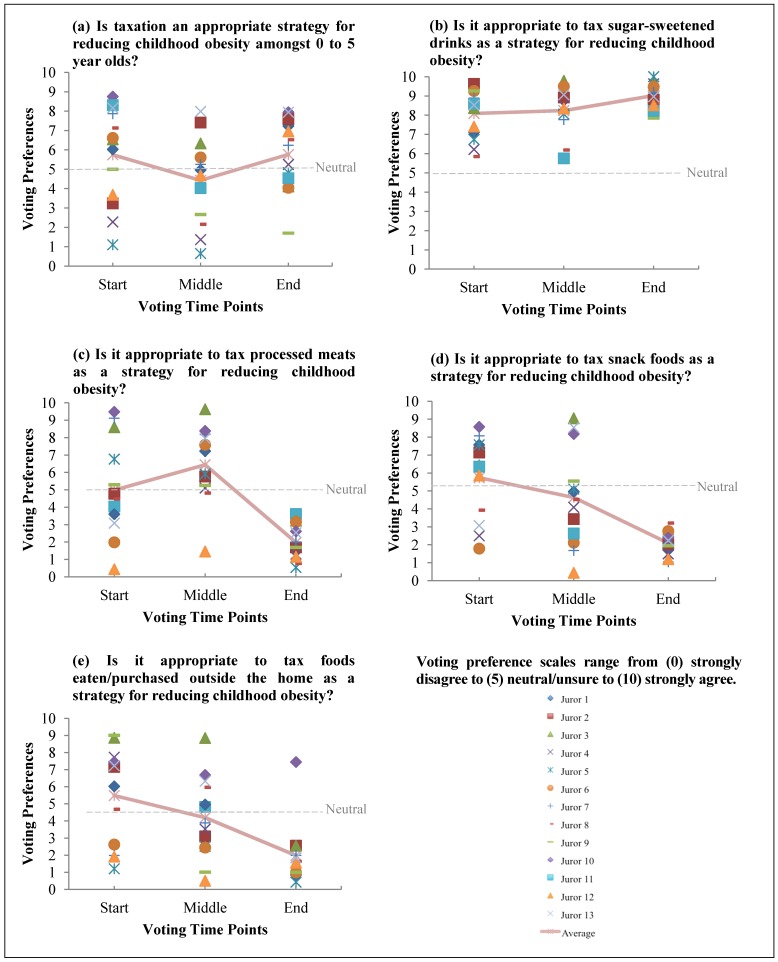
Individual and average voting preferences at three time points for the five questions: (**a**) Taxation. (**b**) Sugar-sweetened drinks. (**c**) Processed meats. (**d**) Snack foods. (**e**) Foods eaten/purchased outside the home.

The individual and average voting preferences of the jurors for each of the questions across the three time points are illustrated in Figure 1 and the verdicts are detailed in the sections below.

#### 3.2.1. Question One: “Is Taxation an Appropriate Strategy for Reducing Childhood Obesity amongst 0 to 5 Year Olds?”

The jurors did not reach a unanimous decision on the overarching question of whether taxation is an appropriate strategy for reducing childhood obesity. Initially, there was wide variation in preferences amongst the jurors; however, by the end of the jury deliberation process half of the jurors were in favour of a tax whilst the remaining jurors were unsure (Figure 1a). Many of the jurors felt that the question was both complex and ambiguous, that is, it was unclear what foods would be taxed. The jurors also stated that the evidence was insufficient to make a decision.

Whilst the jurors felt that a tax is likely to discourage consumption, and therefore reduce obesity rates in children, a number of concerns were raised. Jurors were most concerned about how the revenue that will be raised from the tax would be used. Jurors were unanimous in wanting all revenue generated from the taxation on food and drinks to be directed towards nutrition education and physical activity interventions for children and subsidies for fresh food. They were also concerned about equity issues and whether such a tax would unfairly disadvantage those on low incomes.

#### 3.2.2. Question Two: “Is It Appropriate to Tax Sugar-sweetened Drinks as a Strategy for Reducing Childhood Obesity?”

All jurors strongly agreed that a tax on sugar-sweetened drinks is appropriate. At the start of the Citizens’ Jury process, the jurors were supportive of a tax on sugar-sweetened drinks and their support appeared to increase and consolidate by the end (Figure 1b). Jurors without tertiary education had lower scores at the start than those with College education (6.9 *vs.* 8.8) but they reached consensus by the end (9.1 *vs.* 9.0). The jurors agreed that sugar-sweetened drinks were easily defined and a major contributor to childhood obesity, therefore taxation was a suitable strategy for these products. The jurors felt that the tax needed to be large enough to change consumer behaviour. The majority agreed that a 50% tax was appropriate and some argued for 100% (or doubling of current prices) with most wanting an immediate introduction of taxation. The jurors did not consider equity for those with low incomes to be an issue as such drinks provide little to no health benefits and therefore can be completely removed from the diet.

The jurors were undecided whether diet drinks should be included in this category. It was however agreed that the consumption of such products should not be promoted as there may be health issues associated (e.g., increased dental caries). The jurors felt that packaged unflavoured water should be reduced in price. There were concerns that the full tax may not be passed on to consumers by companies as they may spread the load across all types of sweetened and non-sweetened drinks or there may be unintended consequences such as heavy promotion of diet drinks and non-sweetened drinks with high levels of naturally occurring sugar (e.g., fruit juices). Consequently, in addition to the taxation on sweetened fruit juices, some of the jurors wanted non-sweetened fruit juice (including 100% fruit juice) to also be included in the tax due to the high levels of naturally occurring sugar in such drinks.

#### 3.2.3. Question Three: “Is It Appropriate to Tax Processed Meats as a Strategy for Reducing Childhood Obesity?”

The jurors opposed a tax on processed meats as they felt that the evidence presented did not conclusively indicate that processed meats were a contributing factor to childhood obesity. The jurors highlighted that there are many different types of processed meats and that some types were healthier than others.

As such, the jurors agreed that the types of processed meats that were amenable to taxation were difficult to define and it was, therefore, deemed that taxation was not an appropriate strategy for this category of products. Initially, there was a wide variation in the level of agreement for the introduction of taxation on processed meats with jurors shifting their preference to increased opposition to the tax at the end of the Citizens’ Jury process (Figure 1c).

The jurors felt that more research into the effects of children consuming processed meats is warranted and they strongly believed that the public should be informed of the health consequences of consuming additives commonly found in processed meats such as salt, fat and preservatives. The jurors recommended that the levels of salt and preservatives in products such as processed meats should be reduced and that more nutritional information including the levels of preservatives contained in the product should be included on the food packaging labels. The jurors also believed that education on the effects of consuming processed meats, and processed foods in general, should be given to children from an early age.

#### 3.2.4. Question Four: “Is It Appropriate to Tax Snack Foods as a Strategy for Reducing Childhood Obesity?”

The jurors unanimously agreed that a tax on snack foods was not an appropriate strategy to reduce childhood obesity. There was a wide variation in the level of agreement for a tax on snack foods at the start of the Citizens’ Jury (Figure 1d). The jurors’ opposition to the tax on snack foods appeared to both increase and consolidate over the course of the deliberations. At the end of the Citizens’ Jury, the jurors had a moderately strong opposition to the tax as the category of snack foods is not yet well defined. The jurors believed that the current regulation of snack foods is inadequate and emphasised the need for improvements to be made in regards to nutritional labelling and advertising of snack foods to children. Specifically, jurors recommended that nutritional labelling should include more graphical representations of the sugar content as well as the introduction of the use of a “traffic light” labelling system. The jurors believed that these suggested labelling systems would help consumers to identify healthier food choices. In addition, the jurors believed that such regulation would likely lead to food companies improving the recipes and serving sizes of their snack food products. Furthermore, the jurors stated that they were open to a discussion about the possibility of introducing a tax on the unhealthy snack foods as identified through a ‘traffic light’ labelling system. In regards to advertising of snacks foods, the jurors supported increased regulation of all forms of advertising snack foods to children. This included advertising from media sources to sponsorships of children’s activities to shop displays. The jurors felt that there needs to be increased health messages and advertising of healthy food choices for children.

#### 3.2.5. Question Five: “Is It Appropriate to Tax Food Eaten outside the Home (Purchased outside the Home) as a Strategy for Reducing Childhood Obesity?”

The jury did not support taxation on foods eaten/purchased outside the home. The jurors appeared to have held a wide variation in the levels of support for a tax on these foods throughout the Citizens’ Jury process. Following the final deliberations, however, the jurors reported a moderate to strong opposition of a tax on foods eaten/purchased outside the home with the exception of one juror who moderately supported a tax. The jurors opposed this tax as they believed that the current definition was too broad and difficult to define. Specifically, the jurors highlighted that the definition did not account for healthy foods that are eaten/purchased outside the home and for unhealthy foods that are purchased outside the home and eaten inside the home. The jurors believed that regulation in the form of nutritional labelling was appropriate for this category of foods, supporting a “traffic light” labelling system aimed at helping consumers choose healthier food options. Half of the jurors supported a tax on unhealthy foods eaten/purchased away from home as determined by a ‘traffic light’ labelling system.

### 3.3. Recommendations from the Jurors about Strategies Other Than Taxation

The jurors recognised that food taxation was only one of several strategies necessary to help prevent and reduce childhood obesity in Australia. The jurors proposed a number of strategies that were required in conjunction with taxation to help address childhood obesity as they were reluctant to rely only on taxation noting the importance of informing and supporting healthy consumer decisions. Both education-based strategies and the introduction of regulation were believed to be important in order to help consumers make healthier choices. The types of education-based strategies proposed by the jurors were not specified. The jurors, did, however identify the need for regulation in regards in improved product nutritional labelling and increased access to affordable healthy food options. Specifically, the jurors proposed the introduction of a comprehensive nutritional labelling system, such as “traffic light” labelling, which was regarded as essential in helping consumers identify healthier food options. In addition, the jurors identified the need for clearer labelling of specific nutrients (*i.e.*, sugar, salt, fat, *etc.*) and recommended pictures to be placed on the front of food packages to show the number of teaspoons of the nutrients contained in the product. Furthermore, the jurors believed that access to affordable healthy food options was important to contain the obesity crisis highlighting the need for increased availability of healthier foods within outlets and the need for healthier foods to be cheaper than, or at least the same price as, unhealthy food options. Half of the jurors suggested the regulation of the distribution of the types of food outlets within a geographical area. The jurors concluded with the recommendation that more research in the area of childhood obesity prevention is required in order to address childhood obesity.

### 3.4. Discussion

There was strong support for taxation on sugar-sweetened drinks as an obesity-prevention strategy. This support was evident throughout the deliberations and became stronger by the time the final vote was cast. These results suggest that there may be broad support in the community for the introduction of a tax on sugar-sweetened drinks, particularly if the proposal was accompanied by front-of-pack labelling and increased nutrition education initiatives. This information; similar to what was presented to the jurors; would allow citizens to make informed decisions and judgements about the appropriateness of taxation as a strategy to address childhood obesity.

The jurors considered that the likelihood of public support would be increased if taxation revenue was used to promote healthy eating and/or subsidise healthy food alternatives and if the tax was high enough to change consumption patterns of parents. Taxation on sugar-sweetened drinks has already been implemented in a number of countries [27,28,29]. Evidence has suggested that taxes need to be non-trivial in order to change behaviour [30] and may need to be higher than 10% to have an impact on consumption [29,31]. However, even small taxes that may not affect sales raise significant revenues to fund public health activities [32].

The jury did not support taxation on other categories of food primarily because they included both obesogenic and non-obesogenic options. Jurors acknowledged the difficulties associated with defining healthy and unhealthy foods and the pragmatic implications of this uncertainty for taxation policy. The difficulties associated with taxation of these food categories were highlighted in 2012 when Denmark repealed an unpopular “fat” tax due to public and industry pressure [33].

There was strong support for other types of regulation to assist parents in making healthy choices for their children, indicating that taxation alone was not considered a sufficient strategy. Clear front-of-pack labelling was presented by one of the expert witnesses and was a key recommendation made by the jury, even though food labelling was not the focus of the Citizens’ Jury. In support of this recommendation, recent research has demonstrated that 96% of Queenslanders were unable to identify healthy and unhealthy food because of confusing and misleading labelling practices [34]. The jurors believed that a clear labelling system should be developed and implemented in Australia, if parents are to be supported to make healthy choices for their children.

The recommendations made by this jury support previous strategies identified by public health researchers. They described several conditions that needed to be met for taxation to be successful, including making it clear that the aim of taxation is to reduce consumption and that the revenue generated from the taxation should be allocated to publicly-supported health promotion initiatives. Similar conclusions have been drawn in the literature on this topic [35].

This study is not without limitations. Most notably, there may have been a selection bias in the sample as the jurors were chosen from a pool of individuals that were both interested in participating in the Citizens’ Jury and were available over two days of a weekend. Further, the Citizens’ Jury may not have been representative of the Australian population as all of the jurors resided in the local community. Whilst Citizens’ Juries are a well-accepted approach for engaging the public in decision-making, it is important to note that the findings of the current study are from a small group of jurors and may not reflect the views and opinions of the broader public. Moreover, the method of the presenting information in the form of evidence from expert witnesses is typical of the Citizens’ Jury process however may not be reflective of the usual approach that is adopted when introducing new policies to the general public. Although it is a standard process to present a number of questions to jurors in the Citizens’ Jury [19], it is possible that the presentation of five questions may have yielded different findings than if the jurors had deliberated on a single question. Views from the peak industry groups in Australia (the Australian Food and Grocery Council and the Australian Beverages Council) were only presented from media sources (previous print and radio interviews) as both groups declined participation in the jury process. Finally, the jurors reported that the expert witnesses needed more time to present the information and that the categories of foods (*i.e.*, snack foods; and foods eat/purchased outside the home) were too broad and not well defined.

Future Citizens’ Juries on this topic should ensure that expert witnesses are given adequate time to present sufficient evidence to the jurors, to help jurors make improved decisions and to allow for time for questions. In addition, the questions posed to the jurors should be carefully constructed and clearly defined in order to help jurors deliver meaningful verdicts. Clear definitions of obesogenic foods and drinks are essential to assist policy makers in adopting preventive strategies to address childhood obesity. Many of the recommendations from the jurors were outside the scope of the current Citizens’ Jury. As current policies directed at self-regulation of the food industry in Australia appear to be slow and are not designed to take into account public preferences, it may be useful to conduct a Citizens’ Jury specifically on food labelling and other regulation alternatives.

## 4. Conclusions

In summary, this Citizens’ Jury has suggested that the Australian public would support the introduction of a sugar-sweetened drink taxation. However, the acceptability of such a tax will depend on the way in which proceeds from the taxation revenue are allocated. Importantly, our study has shown that the provision of unbiased expert information about childhood obesity and taxation can increase community support for policy change, even in areas such as taxation on sugar-sweetened drinks that may be sensitive or even divisive. Finally, changes to current food and drink labelling methods were strongly supported by all members of the Citizens’ Jury. These reforms should be considered by government to reduce the future societal costs of obesity.
